# Voltage-dependent anion channels mediated apoptosis in refractory epilepsy

**DOI:** 10.1515/med-2020-0113

**Published:** 2020-08-03

**Authors:** Yan Zhao, Wen-Jing Jiang, Lin Ma, Yan Lin, Xing-Bang Wang

**Affiliations:** Department of Geriatric Medicine, Shandong Key Laboratory of Cardiovascular Proteomics, Qilu Hospital of Shandong University, Jinan, 250012, Shandong Province, China; Department of Neurology, Qilu Hospital of Shandong University, Jinan, 250012, Shandong Province, China

**Keywords:** VDAC, refractory epilepsy, apoptosis

## Abstract

The purpose of this study was to investigate the role of voltage-dependent anion channel (VDAC) in mitochondria-mediated apoptosis of neurons in refractory epilepsy. Western blot analyses were carried out to detect the changes in cytochrome *C*, caspase 9, Bax, and Bcl-2. TUNEL assays were also carried out to investigate cell apoptosis under the upregulation and downregulation of VDAC1 with or without Bax or Bcl-2. VDAC1 induced Bax, Bcl-2, and caspase 9, increasing the release of cytochrome *C*. VDAC1 played an essential role in the apoptotic cell death of refractory epilepsy. It is concluded that VDAC1 plays an important role in refractory epilepsy and could be a possible therapeutic target of anti-epileptic drugs. The current study provides a new understanding of the possible mechanisms of refractory epilepsy.

## 1 Introduction

Epilepsy is one of the most common neurological disorders worldwide. There are approximately 30% of patients with epilepsy who are resistant to anti-epileptic drugs [1]. Refractory epilepsy may lead to more severe physical and psychological comorbidities [2]. Even worse, the risk of sudden unexpected death increases in refractory epilepsy [3]. However, the mechanisms of refractory epilepsy remain unclear.

It has been demonstrated that the abnormal morphology and function of mitochondria occur after seizures, while the abnormal function of mitochondria can also cause seizures [4]. This suggests that there might be a certain correlation between the occurrence of seizures and abnormal mitochondria. Our previous study also found the occurrence of neuronal apoptosis in refractory epilepsy rats [5].

Voltage-dependent anion channel (VDAC) is the main component of the mitochondrial permeability transfer pore to control apoptosis through regulation by direct interaction with the Bcl-2 family proteins [6]. *In vitro*, the electrophysiological study of the liposome has found that anti-apoptotic protein Bcl-xl or Bcl-2 is close to the VDAC. Besides, pro-apoptotic protein Bax or Bak forms a large pore with VDAC, allowing the release of cytochrome *C*. As was reported before, VDAC1 is involved in the process of mitochondria-mediated apoptosis by mediating the release of pro-apoptotic proteins and interacting with anti-apoptotic proteins in tumor cells as well as in other cells. Whether VDAC1 is involved or plays a crucial role in mitochondria-mediated apoptosis in refractory epilepsy has not been clarified. It is necessary to investigate whether VDAC is an essential point in the critical step in the occurrence of apoptosis that happened in refractory epilepsy. We have reported the changes in VDAC in the hippocampus of refractory epilepsy rats [5]. Therefore, we carried out the following study to further explore the correlation of VDAC and apoptosis in the formation of refractory epilepsy, and try to find new targets for the treatment of refractory epilepsy *in vitro*. But what is the most important point through this process? Whether we can modulate the VDAC function through the different expression of upstream factors is unknown.

## 2 Materials and methods

### 2.1 Primary cell culture

Animals were treated humanely to alleviate pain or discomfort, according to protocols approved by the Animal Ethics Committee of Shandong University. Neonatal Wistar rats (postnatal 24 h) were purchased from Shandong University Laboratory Animal Center. Primary hippocampal neurons were prepared from neonatal Wistar rats. The single-cell suspension was transferred to a plastic culture bottle and placed in a CO_2_ incubator (37°C, 5% CO_2_, and 95% humidity). After differential adherence, the neurons with a slow adherence rate were collected and counted by trypan blue staining. The cells were grown in culture plates and observed under an inverted phase contrast microscope.

Immunofluorescence cytochemical staining was used with microtubule associated protein (MAP, a neuron biomarker) and glial fibrillary acidic protein (GFAP, a microglia biomarker) antibodies to detect the purity of primarily neurons.

### 2.2 Establishment of a hippocampal epilepsy model and a refractory epilepsy model *in vitro*

The Sombati method was used to automatically create an epilepsy model [7]. After 10 days of primary culture, the neurons were exposed to zero-Mg^2+^ media for 3 h and then returned to neurobasal media to create the hippocampal epilepsy model. Those cells transfected by rAAV-VDAC, which was constructed as described below, were used as the refractory epilepsy model, selected by phenytoin sodium. The spontaneous epileptiform discharges of both the hippocampal epilepsy model and the refractory epilepsy model were recorded by an electrophysiological instrument, while the signal acquisition and analysis were completed by the pClamp9.2 software.

### 2.3 Construction of plasmids

To increase the efficiency of transfection in primary hippocampal neurons, adenovirus (Ad) vector was used to construct the plasmids. The VDAC1 nucleotides from 157 to 177 (157-AAAGTGAACGGCAGCCTGGAA-177) were selected for the target region of VDAC1 shRNA into GV314 vector (CMV-MCS -3fLAG-SV40-EGFP, GeneChem, Shanghai, China), which was used to reduce VDAC expression. The VDAC1 cDNA, Bax cDNA, and Bcl-2 cDNA were cloned into GV119 vector (hU6-MCS-CMV-EGFP, GeneChem, Shanghai, China) from genomic DNA by polymerase chain reaction (PCR), which was used to increase gene expression. All the Ads were then concentrated, purified, and verified by GeneChem (Shanghai, China).

### 2.4 Real-time quantitative PCR

Total RNA was extracted from cells using the TRIzol Reagent and purified with Direct-zol RNA kits (Invitrogen). cDNA was synthesized using the SuperScript III First-Strand Synthesis System for RT-PCR kit (Invitrogen). The transcript cDNA was amplified by Real-time PCR using a Takara’s Perfect Real-Time PCR kit (Takara Bio, Otsu, Japan) and Applied Biosystems™ 7900 Real-Time PCR detection system (Thermo Fisher Scientific Inc., Waltham, MA, USA). All primers were purchased from Invitrogen, VDAC1 (F:5′-CGGGATCCATGATAAAACTTGATTTGAAAACG-3′, R:5′-GCGGCCGCTTATGCTTGAAATTCCAGTCC-3′) and β-actin (F:5′-CACGATGGAGGGGCCGGACTCATC-3′, R:5′-TAAAGACCTCTATGCCAACACAGT-3′). β-Actin was used as an internal control to normalize sample differences.

### 2.5 Detection of apoptosis by the TUNEL assay

Apoptotic cells were analyzed and detected by the TUNEL assay kit-FITC (Abcam), according to the recommended protocol. The cells were fixed with formaldehyde on ice, washed, rinsed, incubated, and analyzed with a fluorescence microscope.

### 2.6 Western blot

Mitochondrial and cytoplasmic fractions from cultured cells were isolated with Mitochondria/Cytosol Fractionation Kit from Abcam, according to the recommended protocol.

Equal quantities of proteins were separated by 12% SDS-polyacrylamide gels and transferred onto PVDF membranes (Thermo Fisher Scientific Inc., Waltham, MA, USA), and immunoblotted with specific primary antibodies. After incubation with the corresponding secondary antibodies, proteins were detected by SuperSignal® West Pico Chemiluminescent Substrate (Thermo Fisher Scientific Inc., Waltham, MA, USA). Quantification was performed via ImageJ software (NIH, Bethesda, Maryland, USA).

The primary antibodies used in this study were as follows: Mouse Anti-VDAC1 antibody (ab14734, Abcam), Rabbit Anti-Bax antibody (ab32503, Abcam), Rabbit Anti-Bcl-2 antibody (ab7973, Abcam), Mouse Anti-cytochrome *C* antibody (ab110325, Abcam), and Rabbit Anti-Caspase-9 antibody (ab32539, Abcam). β-Actin (ab8226, Abcam) served as a loading control.

## 3 Statistical analysis

All experiments were repeated three times. Data were expressed as means ± S.D. in this study. Statistical evaluation was carried out using Student’s *t*-test and *p* value less than 0.05 (*) was considered statistically significant. All statistical analyses were conducted by SPSS 23.0.

## 4 Results

### 4.1 VDAC1 induced cell apoptosis in refractory epilepsy

**Figure 1:**
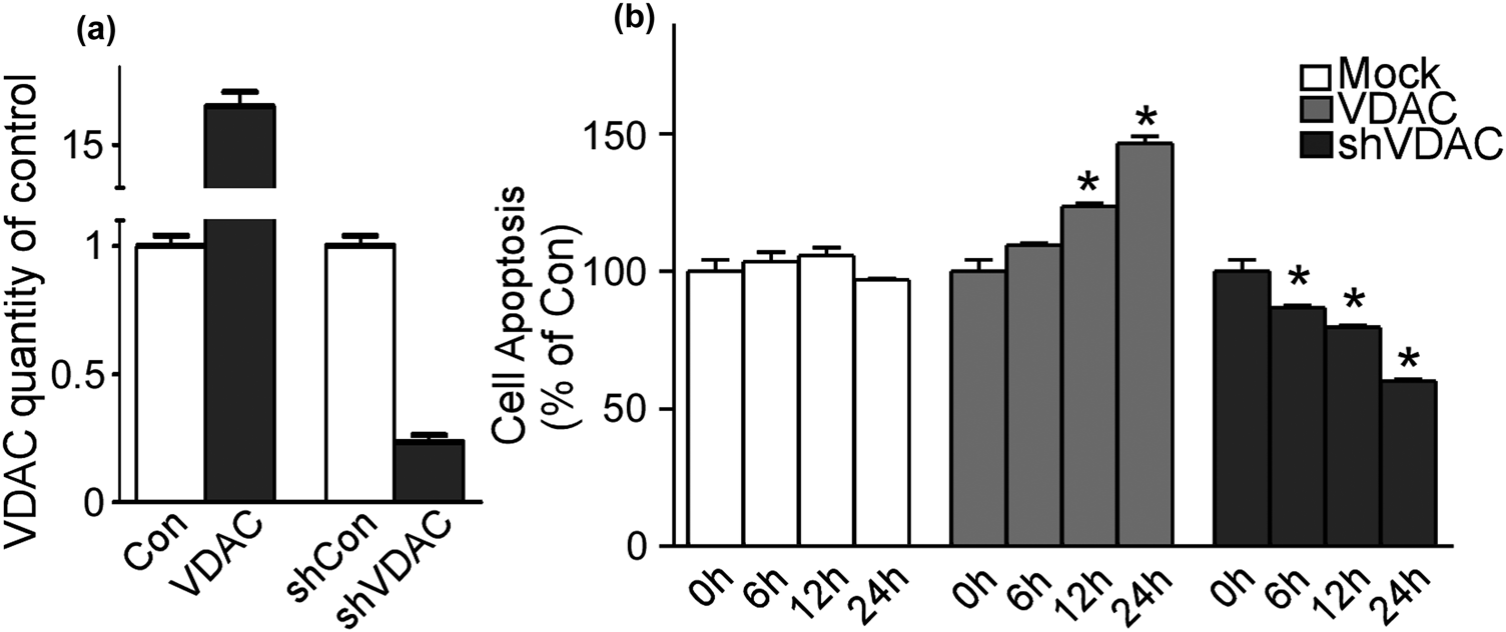
VDAC1 induced cell apoptosis in refractory epilepsy. (a) The expression of VDAC1 validated by PCR at upregulation and downregulation. (b) Cell apoptosis at different time points detected by the TUNEL assay. Values represent means ± SD, *n* = 3. *P* < 0.05 by Student’s *t*-test.

Spontaneous epileptic discharge models of hippocampal neurons were established using the Sombati method. VDAC1-overexpressed neurons were constructed to mimic the condition of refractory epilepsy, detected and confirmed by phenytoin sodium. VDAC1 was upregulated and downregulated to detect the role of VDAC1 in the following process. PCR was carried out to validate the transfection efficiency of upregulation and downregulation of VDAC1 (shVDAC1) (Figure 1a).

Cell apoptosis was detected in epilepsy neurons, refractory epilepsy neurons with the gain function of VDAC1, and refractory epilepsy neurons with the loss function of VDAC1 using TUNEL labeling. With upregulation of VDAC1, cell death significantly increased up to 146.5 ± 5.1% after 24 h (*p* < 0.05) (Figure 1b). On the contrary, with downregulation of VDAC1, cell death decreased to 60.2 ± 1.4% after 24 h (*p* < 0.05) (Figure 1b). These results showed that the mitochondria protein, VDAC1, induced apoptotic cell death in refractory epilepsy and downregulation VDAC1 could reverse the process.

### 4.2 VDAC1 increased the release of cytochrome *C* into cytosol and upregulation of caspase 9 in refractory epilepsy

**Figure 2:**
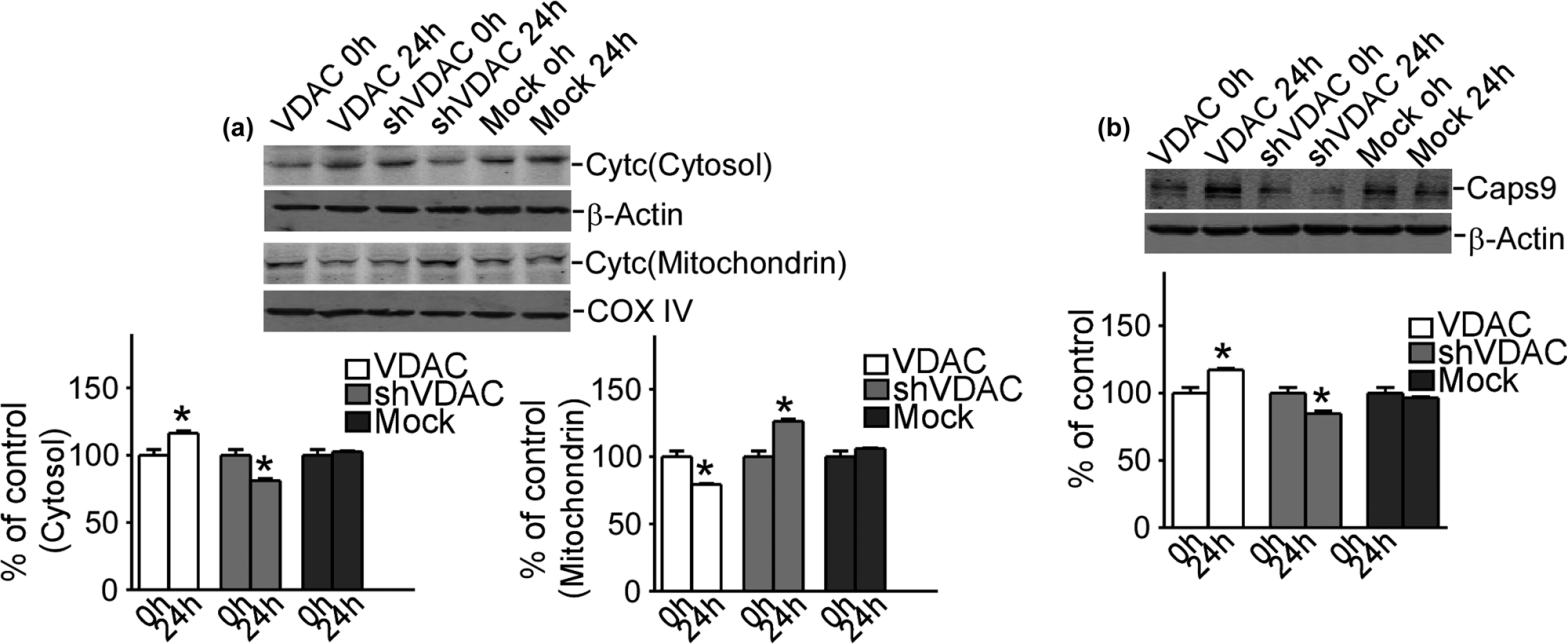
VDAC1 increased the release of cytochrome *C* and expression of caspase 9. (a) The changes of Cyt *C* in mitochondria and cytosol with upregulation and downregulation of VDAC1. (b) The changes of Caps9 with upregulation and downregulation of VDAC1. Cyt *C* means cytochrome *C*, Caps9 means caspase 9. Values represent means ± SD, *n* = 3. *P* < 0.05 by Student’s *t*-test.

Western blot was used to detect apoptosis-related cytochrome *C* and caspase 9 expression levels to clarify that these two factors change along with VDAC1 in refractory epilepsy. Apoptosis was obvious in 24 h, so this point was chosen to compare the differences of these factors (Figure 1b). There was no difference in epilepsy neurons, but upregulation of VDAC1 in refractory epilepsy neurons increased the release of cytochrome *C* from mitochondria to cytosol (77.9 ± 2.31% and 116.7 ± 2.54%, respectively, *P* < 0.05) (Figure 2a). Also during downregulation of VDAC1 in refractory epilepsy neurons, the release of cytochrome *C* was blocked (mitochondria 126.2 ± 1.97% and cytosol 80.3 ± 3.19%, respectively, *P* < 0.05) (Figure 2a).

Accompanied by the release of cytochrome *C* into cytosol, caspase 9 increased obviously in refractory epilepsy neurons with the gain function of VDAC1 (117.6 ± 1.75%, *P* < 0.05) and decreased in refractory epilepsy neurons with the loss function of VDAC1 (84.7 ± 2.66%, *P* < 0.05) (Figure 2b). These results showed that VDAC1 played an essential role in the release of cytochrome *C* and increase of caspase 9 in refractory epilepsy. It was desired to detect the changes of apoptotic proteins and anti-apoptotic proteins in the process.

### 4.3 VDAC1 induced the expression of Bax and Bcl-2 in refractory epilepsy

**Figure 3:**
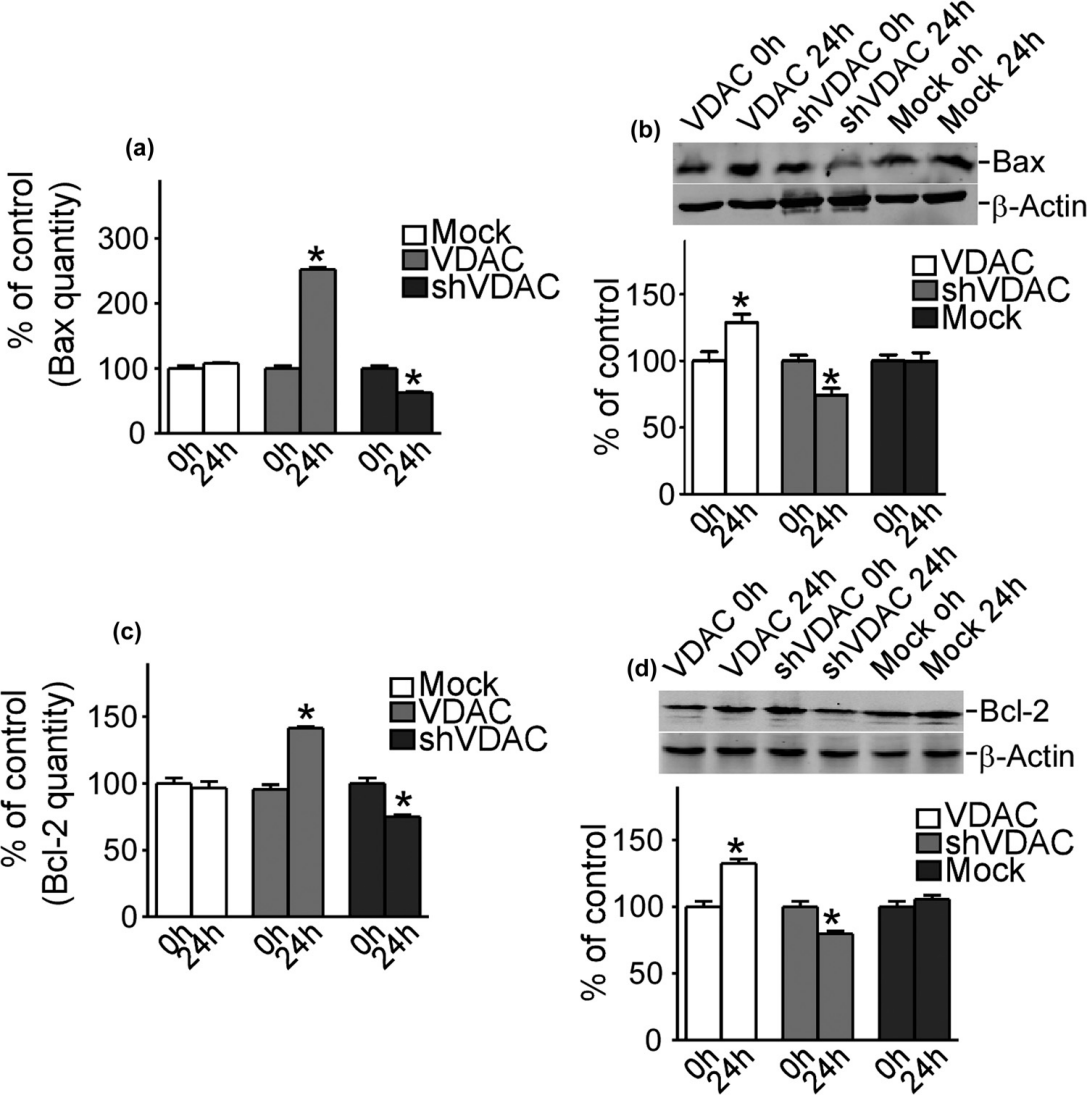
VDAC1 increased the expression of Bax and Bcl-2. (a and b) The mRNA and protein levels of Bax with upregulation of VDAC1. (c and d) The mRNA and protein levels of Bcl-2 with upregulation of VDAC1. Values represent means ± SD, *n* = 3. *P* < 0.05 by Student’s *t*-test.

In this part, it was aimed to investigate the changes of apoptosis-related proteins, Bax and Bcl-2, in refractory epilepsy. Bax and Bcl-2 were detected by PCR and western blot in 0 and 24 h. Both the mRNA (Figure 3a) and protein (Figure 3b) levels of Bax were significantly increased in refractory epilepsy neurons with the gain function of VDAC1 (252.5% ± 6.5% and 151.6 ± 6.47%, respectively, *P* < 0.05). On the contrary, both mRNA and protein levels of Bax were significantly inhibited in refractory epilepsy neurons with the gain function of VDAC1 (68.9% ± 0.9% and 64.1% ± 8.2%, respectively, *P* < 0.05).

The mRNA (Figure 3c) and protein (Figure 3d) levels of Bcl-2 changed similarly to those of Bax and both levels obviously increased in refractory epilepsy neurons with the gain function of VDAC1 (146.5% ± 5.1% and 132.8% ± 3.33%, respectively, *P* < 0.05) and decreased in refractory epilepsy neurons with the gain function of VDAC1 (60.2 ± 1.4% and 79.6% ± 1.21%, respectively, *P* < 0.05). These results showed that the expression of Bax and Bcl-2 could be regulated by the VDAC1 in refractory epilepsy.

### 4.4 Roles of Bax and Bcl-2 on the effect of VDAC1 in refractory epilepsy

We have clarified that expressions of Bax and Bcl-2 were both increased in refractory epilepsy, as described above. It is necessary to further investigate the underlying mechanisms of these two factors in this refractory epilepsy model. Therefore, these two factors were upregulated to enlarge the effects in refractory epilepsy neurons. To start with, Ads were used to achieve the upregulation of Bax and Bcl-2. Then TUNEL labeling was used to measure cell apoptosis and western blot was used to detect changes in expression levels of cytochrome *C* and caspase 9.

**Figure 4:**
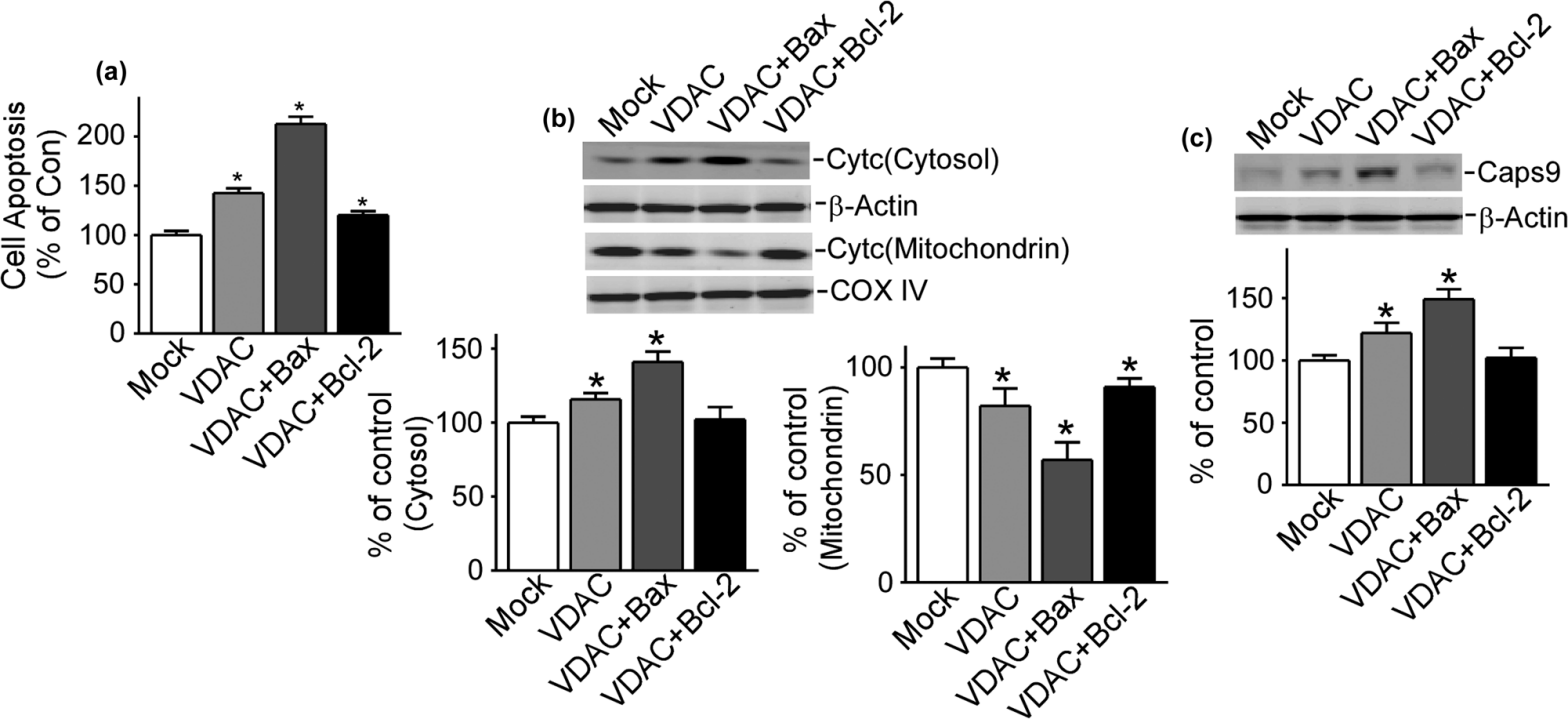
VDAC1 related to Bax and Bcl-2 in refractory epilepsy. (a–c) Cell apoptosis, the release of Cyt *C*, and the expression of Caps9 with upregulation of VDAC1 and Bax, or VDAC1 and Bcl-2. Cyt *C* means cytochrome *C*, Caps9 means caspase 9. Values represent means ± SD, *n* = 3. *P* < 0.05 by Student’s *t*-test.

With upregulation of Bax in refractory epilepsy neurons with the gain function of VDAC1, cell apoptosis significantly increased up to 212.5% ± 15.0% (*P* < 0.05) combined with refractory epilepsy neurons (142.5% ± 9.6%, *P* < 0.05) (Figure 4a). On the contrary, with upregulation of Bcl-2 in refractory epilepsy neurons with the gain function of VDAC1, cell apoptosis decreased to 120.0% ± 8.2% (*P* < 0.05) combined with refractory epilepsy neurons (Figure 4a).

Cytochrome *C* and caspase 9 were then investigated in the apoptosis pathway to better clarify this issue. Upregulation of Bax in refractory epilepsy neurons with the gain function of VDAC1 promoted the release of cytochrome *C* from mitochondria to cytosol (57.0% ± 16.3% and 149.0% ± 16.3%, respectively, *P* < 0.05) combined with refractory epilepsy neurons (82.0% ± 12.1% and 122.0% ± 15.4%, respectively, *P* < 0.05) (Figure 4b). However, with upregulation of Bcl-2 in refractory epilepsy neurons with the gain function of VDAC1, the release of cytochrome *C* was inhibited (mitochondria 90.8% ± 8.2 and cytosol 102.0% ± 16.3%, respectively, *P* < 0.05) combined with refractory epilepsy neurons (Figure 4b).

The expression of caspase 9 was also obviously increased with upregulation of Bax in refractory epilepsy neurons with the gain function of VDAC1 (141.0% ± 14.1%, *P* < 0.05), but decreased with upregulation of Bcl-2, when combined with refractory epilepsy neurons (115.8% ± 8.3%, *P* < 0.05) (Figure 4c). It could be seen that in refractory epilepsy neurons, the pro-apoptotic protein Bax and anti-apoptotic protein Bcl-2 played their collaborative roles in apoptosis, the release of cytochrome *C* to cytosol and increase of caspase 9 were promoted by Bax and inhibited by Bcl-2 in refractory epilepsy neurons, what’s more, all effects are related to VDAC1.

### 4.5 VDAC1 regulates the effects caused by Bax and Bcl-2 in epilepsy

As described above, both Bax and Bcl-2 played an important role in apoptosis in refractory epilepsy. We wondered whether those two factors acted in the same way in epilepsy and whether they could be regulated by downregulation of VDAC1. Consequently, in the following part, western blot was carried out to detect expression changes in the process.

**Figure 5:**
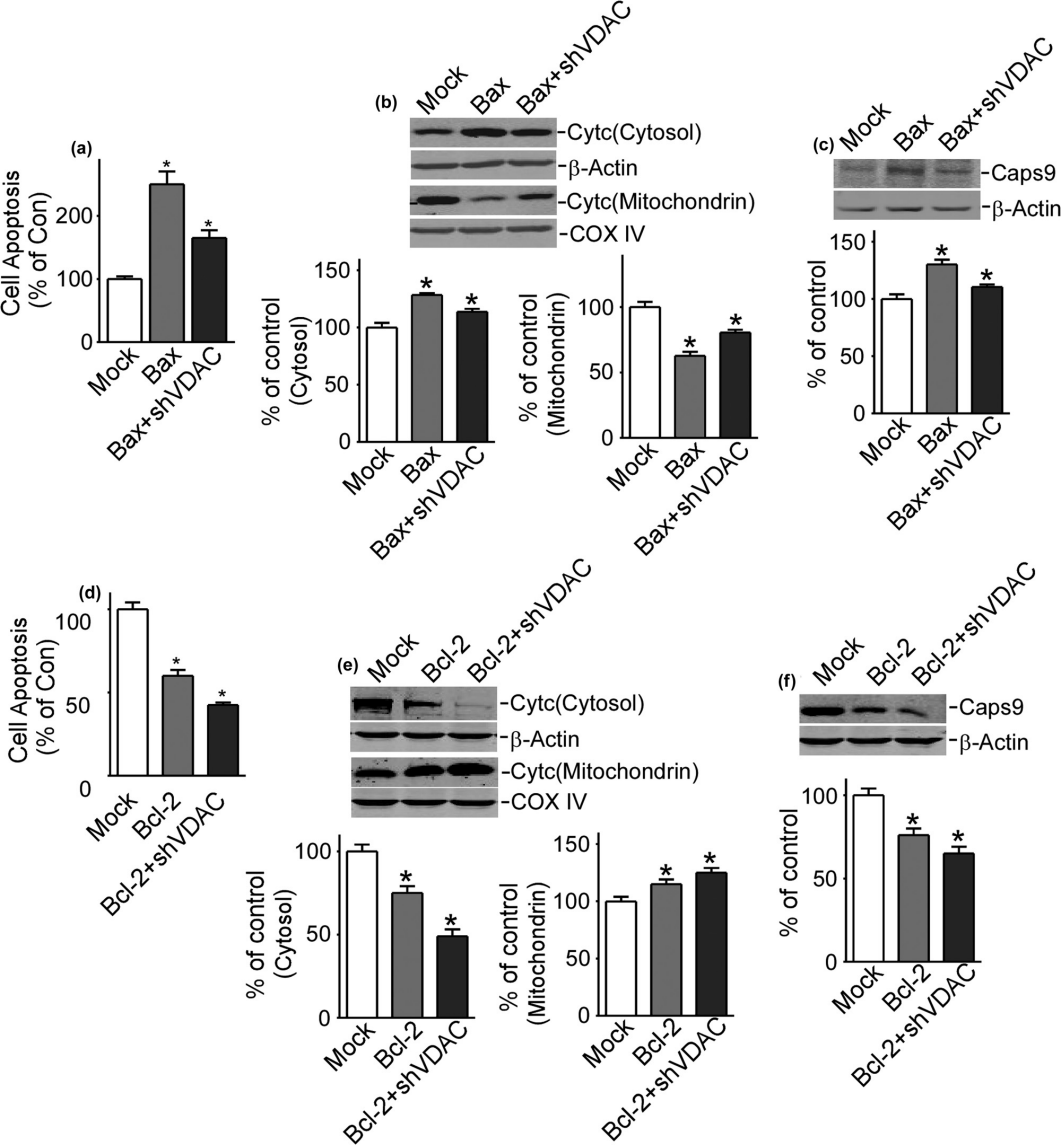
VDAC1 regulates the effects caused by Bax and Bcl-2 in epilepsy. (a–c) Cell apoptosis, the release of Cyt *C*, and the expression of Caps9 with upregulation of Bax, or upregulation of Bax and downregulation of VDAC1. (d–f). Cell apoptosis, the release of cytochrome *C*, and the expression of caspase 9 with upregulation of Bcl-2, or upregulation of Bcl-2 and downregulation of VDAC1. Cytc means cytochrome *C*, Caps9 means caspase 9. Values represent means ± SD, *n* = 3. *P* < 0.05 by Student’s *t*-test.

When Bax was upregulated in refractory epilepsy neurons, cell apoptosis significantly increased up to 250.0% ± 40.8% (*P* < 0.05) (Figure 5a), suggesting Bax as a pro-apoptotic protein in this epilepsy model. Then when Bax was upregulated in refractory epilepsy neurons with the loss function of VDAC1, the apoptosis decreased to 165.0% ± 23.8% (*P* < 0.05) (Figure 5a). Next, changes of cytochrome *C* and caspase 9 were continually investigated in the following process. Bax promoted the release of cytochrome *C* from mitochondria to cytosol in refractory epilepsy neurons (62.8% ± 6.1% and 128.5% ± 3.1%, respectively), which was also inhibited in refractory epilepsy neurons with the loss function of VDAC1 (mitochondria 80.5% ± 4.2% and cytosol 113.8% ± 5.1%, respectively, *P* < 0.05) (Figure 5b). Accompanied by the release of cytochrome *C*, caspase 9 obviously increased at the same time in refractory epilepsy neurons (130.3% ± 8.2%, *P* < 0.05) (Figure 5c), which decreased in refractory epilepsy neurons with the loss function of VDAC1 (110.5% ± 4.2%, *P* < 0.05). Therefore, Bax induced the cell apoptosis, release of cytochrome *C*, and increase of caspase 9 in epilepsy, which could be inhibited by VDAC1 downregulation.

Bcl-2 upregulation in refractory epilepsy neurons significantly decreased cell apoptosis to 60.0% ± 7.1% (*P* < 0.05), which showed that Bcl-2 acted as an anti-apoptotic protein in this epilepsy model. The apoptotic process was further inhibited in refractory epilepsy neurons with the loss function of VDAC1 (42.5% ± 2.9%, *P* < 0.05) (Figure 5d). Bcl-2 inhibited the release of cytochrome *C* from mitochondria to cytosol in refractory epilepsy neurons (115.0% ± 8.2% and 75.0% ± 8.2%, respectively, *P* < 0.05), which was strengthened in refractory epilepsy neurons with the loss function of VDAC1 (mitochondria 125.0% ± 8.2% and cytosol 49.0% ± 8.2%, respectively, *P* < 0.05) (Figure 5e). Caspase 9 was decreased obviously (76.0% ± 8.2%, *P* < 0.05) by Bcl-2 in refractory epilepsy neurons and became even more significant in accordance with refractory epilepsy neurons with the loss function of VDAC1 (65.0% ± 8.2%) (Figure 5f). In this part, we could see that Bcl-2 inhibited the cell apoptosis, release of cytochrome *C*, and activation of caspase 9 in epilepsy, which could be strengthened by VDAC1 downregulation.

## 5 Discussion

More and more studies have found that apoptosis plays an important role in tumor resistance against chemotherapy [8,9]. We wonder whether mitochondria-mediated apoptosis is involved in the formation of refractory epilepsy. Therefore, we try to imitate the refractory epileptic status and detect the functions of VDAC1 in the process.

VDAC is an integral membrane protein, mediating the trafficking of tiny molecules between mitochondria and cytosol, and regulating metabolic signals as well as other pathways [10,11]. There are three VDAC isoforms identified in mammals, includingVDAC1, VDAC2, and VDAC3 [10]. Increasing evidence point out that VDAC1 plays an essential role in mitochondria-mediated apoptosis, which is implicated in neurodegenerative disorders [12,13,14] as well as in cancer [10,15]. Though there are some studies concerning the connection between apoptosis and epilepsy, little is known about the role of VDAC1 in epilepsy as well as in refractory epilepsy [16]. We previously observed protein expressions in the refractory epilepsy model created in electrical amygdala-kindled rats through the method of two-dimensional gel electrophoresis and matrix-assisted laser desorption/ionization time of flight [5]. Based on these results, we found the changes of mitochondrial VDAC1 level with a 2.82-fold increase in hippocampus of pharmacoresistant rats, compared with those in hippocampus of non-pharmacoresistant rats [5].

There are many refractory epilepsy models *in vivo* and *in vitro* [17,18,19]. Although *in vivo* models are good at reproducing the complexity of a living being, it might be influenced by other factors. Using *in vitro* models would possibly decrease those influence. Consequently, we used hippocampal epileptic neurons created by the Sombati method, which is well recognized, easily operated, and less influenced by unexpected factors [7]. Owing to the lack of a refractory epilepsy cell model, VDAC1-overexpressing cells were constructed to mimic this condition, which would been screened by phenytoin sodium and validated by the patch clamp [17,18].

The work presented here demonstrates the effects of VDAC1 on mitochondria-mediated apoptosis in refractory epilepsy neurons. Downstream apoptotic proteins, such as cytochrome *C* and caspase 9, are regulated by the VDAC1. The results indicate that apoptosis is induced by the increased release of cytochrome *C* into cytosol and expression of caspase 9. Also, we found that the downregulation of VDAC1 inhibited cell apoptosis. These findings extend our previous observations on the upregulation of VDAC1 in refractory epilepsy rats [5]. It is an important finding and we got to speculate whether VDAC1 is indispensable in refractory epilepsy. Further investigation on the mechanism is of importance to better understand refractory epilepsy.

In our study, both Bax and Bcl-2 increased with the overexpression of VDAC1 in refractory epilepsy. Bax and Bcl-2 are both Bcl-2 family proteins, which are key factors in regulating intrinsic apoptosis [20]. Specifically, Bax is a pro-apoptotic protein, while Bcl-2 is an anti-apoptotic protein [20]. Consequently, the results seem fairly paradoxical because these two factors play opposite roles in intrinsic apoptosis. We know that cells show widely different sensitivities to apoptosis. The expression levels of Bax and Bcl-2 might be variable under different conditions and stimulations, developmental stages, and so on. We assume that there might be two possible mechanisms contributing to the increase of Bcl-2 in this study: (1) Bcl-2 acts as an anti-apoptotic activator to bind receptors on mitochondria in order to inhibit the binding of pro-apoptotic activators with receptors [21]. When saturated or absent, Bcl-2 increases to bind more receptors to antagonize pro-apoptotic chains. The upregulation of Bcl-2 with DNA damage was found in Alzheimer’s disease, possibly as a protective mechanism [21]. (2) As post-mitotic neurons with less replaceable cells, their apoptosis-regulating proteins are apt to be downregulated to resist apoptosis [22,23]. Under some circumstances, apoptosis might be induced, while anti-apoptotic proteins like Bcl-2 might be upregulated to maintain the survival of neurons.

Although Bax and Bcl-2 were both increased in our study, cell apoptosis still occurred. It indicates that pro-apoptotic effects overwhelmed anti-apoptotic effects in this model. However, the real environment in cells is very complicated and the balance between Bax and Bcl-2 in the apoptosis pathway still needs to be elucidated.

In view of the increase of Bax and Bcl-2 in refractory epilepsy, we should investigate the exact role and degree of these two factors in apoptosis. We found that apoptosis was induced followed by the release of cytochrome *C* into cytosol and activation of caspase 9, in the detection of Bax. Bcl-2, a promising anti-apoptotic protein, inhibited the process of apoptosis. The roles of Bax and Bcl-2 in mitochondria-mediated apoptosis in refractory epilepsy are well confirmed.

Apart from that in refractory epilepsy, we also detected the role of VDAC1 in epilepsy. VDAC1 regulated the pro-apoptotic effects of Bax and Bcl-2 in epilepsy. It has been investigated that Bax/Bak can bind VDAC1 via direct interaction and modulate its activity [24]. Although apoptosis has been induced by Bax, the downregulation of VDAC1 may decrease the binding of Bax with VDAC1, which inhibits the downstream apoptotic process with deficient binding. The anti-apoptotic protein Bcl-2 can close VDAC1 on mitochondria [25]. The downregulation of VDAC1 makes Bcl-2 less likely saturated with VDAC1. This will enhance the anti-apoptotic effects of Bcl-2.

This report demonstrates that VDAC1 plays an important and key role in mitochondria-mediated apoptosis in refractory epilepsy. It provides a new possible mechanism of apoptosis that happened in refractory epilepsy. Also, drugs that inhibit apoptotic pathways are often developed in tumors [26,27]. Our study might offer a possible therapeutic target of anti-epileptic drugs. However, more *in vivo* studies on animals are needed to further investigate the consequence of VDAC1. Also, homozygote VDAC1 knockout (VDAC1−/−) mice can be used in future research studies.
